# Clinical Importance of Potential Genetic Determinants Affecting Propofol Pharmacokinetics and Pharmacodynamics

**DOI:** 10.3389/fmed.2022.809393

**Published:** 2022-02-28

**Authors:** Ivana Budic, Tatjana Jevtovic Stoimenov, Dimitrije Pavlovic, Vesna Marjanovic, Ivona Djordjevic, Marija Stevic, Dusica Simic

**Affiliations:** ^1^Department of Surgery and Anesthesiology, Faculty of Medicine, University of Niš, Niš, Serbia; ^2^Clinic for Anesthesiology and Intensive Therapy, University Clinical Center Nis, Niš, Serbia; ^3^Institute of Biochemistry, Faculty of Medicine, University of Niš, Niš, Serbia; ^4^Clinic for Plastic and Reconstructive Surgery, University Clinical Centre Nis, Niš, Serbia; ^5^Clinic for Pediatric Surgery and Orthopedics, University Clinical Center Nis, Niš, Serbia; ^6^Department of Surgery and Anesthesiology, Faculty of Medicine, University of Belgrade, Belgrade, Serbia; ^7^Department of Anesthesiology and Intensive Therapy, University Children’s Hospital, Belgrade, Serbia

**Keywords:** propofol, pharmacokinetics, pharmacodynamics, gene, polymorphism

## Abstract

Interindividual variability in response to drugs used in anesthesia has long been considered the rule, not the exception. It is important to mention that in anesthesiology, the variability in response to drugs is multifactorial, i.e., genetic and environmental factors interact with each other and thus affect the metabolism, efficacy, and side effects of drugs. Propofol (2,6-diisopropylphenol) is the most common intravenous anesthetic used in modern medicine. Individual differences in genetic factors [single nucleotide polymorphisms (SNPs)] in the genes encoding metabolic enzymes, molecular transporters, and molecular binding sites of propofol can be responsible for susceptibility to propofol effects. The objective of this review (through the analysis of published research) was to systematize the influence of gene polymorphisms on the pharmacokinetics and pharmacodynamics of propofol, to explain whether and to what extent the gene profile has an impact on variations observed in the clinical response to propofol, and to estimate the benefit of genotyping in anesthesiology. Despite the fact that there has been a considerable advance in this type of research in recent years, which has been largely limited to one or a group of genes, interindividual differences in propofol pharmacokinetics and pharmacodynamics may be best explained by the contribution of multiple pathways and need to be further investigated.

## Introduction

One of the most challenging areas of research in clinical pharmacology, pharmacy, pharmacoepidemiology, and especially pharmacogenetics is the attempt to understand why individuals respond differently to drug therapy. Problems with drug therapy can be divided into two main categories. The first problem is that the drugs are not equally effective in all patients. If it were possible to predict the efficacy of the drug in advance, interindividual variation of the drug would be avoided in patients in whom the drug does not work enough, and at the same time the costs would be reduced. Another major therapeutic problem is the occurrence of adverse drug events (ADEs), which is especially important in the fields of medicine where drugs of small therapeutic range are used, among which are anesthesiology and intensive care.

Several evident examples of unfavorable outcomes from perioperative drugs are well-known (e.g., malignant hyperthermia, prolonged apnea, respiratory depression, and insufficient analgesia), leading to a better knowledge of the genetic susceptibilities behind these problems. Despite this, systematic genetic screening prior to surgery to determine drug risk is not currently common practice (1).

Adverse drug events or overdose are responsible for nearly half of anesthesia-related deaths (2), and one out of every 20 perioperative medication doses results in an unanticipated ADE or a medication error (3). Medications such as sedative-hypnotics, inhalation and intravenous anesthetics, analgesics, and cardiovascular drugs, among others, are frequently used in perioperative treatment. It should be noted that patients have generally never received these drugs before. For this reason, pharmacogenomics may play a part in the anesthesiologist’s preoperative evaluation since it allows for individualized anesthetic plans.

In recent years, rapid breakthroughs in molecular biology and the Human Genome Project have resulted in the discovery of millions of new polymorphisms (4). The Clinical Pharmacogenetics Implementation Consortium (CPIC) was established in 2009 to provide a framework for understanding the levels of evidence required for pharmacogenetics to be incorporated into clinical practice, as well as to address the need to provide very specific guidance to clinicians and laboratories in order to ensure that pharmacogenetic tests are used wisely (5, 6).

To explain heterogeneity in drug therapy responses, anesthesiologists and other clinicians have focused on genetic variability that affects drug metabolizing enzymes. Many other essential proteins, including as transporter proteins and receptors, are now known to be affected by genetic variability (7).

Propofol is a short-acting intravenous anesthetic that is commonly used to induce and maintain general anesthesia as well as procedural sedation. Polymorphisms in cytochrome P450 (CYP) isoforms and UDP-glucuronosyltransferase (UGT), as well as drugs administered concurrently, could cause unpredictable interindividual variability of propofol pharmacokinetics and pharmacodynamics with forensic and clinically relevant adverse outcomes e.g., respiratory and cardiac depression, “propofol-related infusion syndrome – PRIS” (8).

## Methods

The electronic search for this narrative review included three databases, PubMed, EMBASE, and Google Scholar, and used search terms: “propofol,” “pharmacokinetics,” “pharmacodynamics,” “gene,” and “polymorphisms.” The inclusion criteria were: articles for which full text was available, studies conducted in adults and children. The exclusion criteria were: articles that were not in English, or were gray literature. From the articles retrieved in the first round of search, additional references were identified by a manual search among the cited references. The search was limited to papers published between 2000 and 2021, and 66 papers were found to be eligible for study.

## Propofol – Structure and Physical Properties

Propofol is by structure isopropyl phenol which is insoluble in water. Chemically it represents 2,6-diisopropylphenol. In commercial preparations, it is packaged in the form of an emulsion containing soybean oil, glycerol and egg lecithin. Propofol is a characteristic viscous, milky-white emulsion, called “milk of anesthesia.” The lipoid emulsion of propofol possesses antioxidant properties when observed *in vivo* and *in vitro*. This property of propofol originates from its chemical nature because it has a structure similar to phenolic antioxidants, such as endogenous alpha-tocopherol (vitamin E) (9). Hence, it is particularly effective in preventing damage caused by ischemia and reperfusion (10, 11). Propofol increases expression of antioxidants, decreases production of reactive oxygen species (ROS), and thus alleviates DNA damage and cell death (12).

## Pharmacokinetics of Propofol

Propofol’s pharmacokinetics (PK) have been thoroughly investigated (13, 14). Propofol PK is commonly described using a three-compartmental model: a large central compartment, a peripheral compartment with lower perfusion (lean tissues), and a deep compartment with low perfusion (fat). Rapid start of action at the brain is ensured by high lipophilicity, and rapid redistribution from the central to peripheral compartment promotes rapid anesthetic action offset (15). Fat compartments at the periphery act as reservoirs, and redistribution from these compartments to the central compartment might take a long time, especially in obese and severely ill patients (16, 17).

Propofol should only be used intravenously. Because of its bitter taste and low oral bioavailability caused by a high first-pass effect and a high hepatic extraction rate (90 percent), it is not suited for enteral or other routes of administration. Propofol is significantly bound to plasma proteins (mostly albumin) and erythrocytes after intravenous administration.

Propofol crosses the blood–brain barrier (BBB) quickly and causes unconsciousness (sometimes in less than the time it takes for a drug to pass through the circulation once). Because of the rapid initial distribution, the period to offset clinical effects after a single bolus or brief infusion is short. Because redistribution of drug from the slow compartment is slower compared to the rates of metabolism and excretion, the offset of clinical effects is nevertheless relatively fast compared to other intravenous hypnotics even after prolonged treatment (18).

**FIGURE 1:**
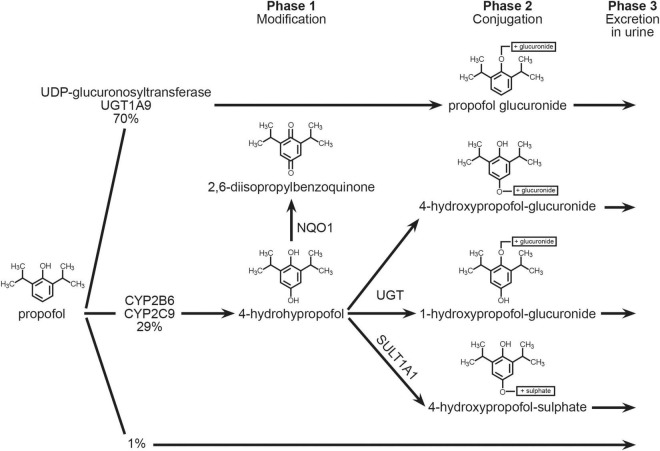
Metabolic pathway of propofol.

Propofol is metabolized in the liver to a multitude of metabolites, most of which are excreted in the urine. The biotransformation can occur in a variety of ways (Figure 1). The small intestines are also metabolically active, with an extraction ratio of 24% (19). The role of the lungs is still being discussed; some studies imply that the lungs play an active role (20), while others do not (21), or that the lungs are only a temporary propofol reservoir that later releases propofol from binding sites back into circulation (22). Furthermore, the kidneys likely account for about one-third of total body propofol clearance in patients undergoing cardiac surgery (21). Only about 0.3 percent of propofol administered is excreted unchanged. Propofol can also be exhaled.

The UDP-glucuronosyltransferase gene encoded by UGT1A9 (UDP glucuronosyltransferase 1 family, polypeptide A9, MIM 606434) is responsible for the majority of propofol’s metabolism (about 70%) into propofol glucuronide (PG). The enzymes coded by the CYP2B6 (MIM 123930) and CYP2C9 (MIM 601130) genes, as well as the SULT1A (MIM 171150) and NQO1 (MIM 125860) genes, execute an alternate pathway of propofol biotransformation (about 29 percent) (23, 24). The cytochrome P450 enzymes (CYP2B6 and CYP2C9) are responsible for the formation of a hydroxyl derivative propofol-4-hydroxypropophol, which can further be transformed into 4-hydroxypropophol-1-ObD-glucuronide (Q1G) and 4-hydroxypropophol-4-ObD-glucuronide (Q4G). About 70–90% of propofol is eliminated by urine in the form of the glucuronide metabolite. It is possible that single nucleotide polymorphisms in the genes encoding these enzymes are responsible for the formation of individual variables of propofol metabolic products, resulting in unpredictable effects of standard anesthetic doses as well as prolonged waking time (recovery) from anesthesia (25).

### CYP2C9 Gene

The enzyme CYP2C9 is a hemoprotein that participates in the first phase of biotransformation of xenobiotics and endogenous molecules and belongs to the large family of CYP2C genes. It makes up 15% of the total metabolism of the first phase of biotransformation. The gene for CYP2C9 is located on chromosome 10q24 and consists of 9 exons. More than 60 alleles of the gene for this enzyme are known. The most common polymorphic allele is CYP2C9*2 (rs1799853, 430C > T). This protein is characterized by weak metabolic activity. The gene encoding this enzyme is polymorphic, which is important for clinical practice because the enzyme CYP2C9 participates in the metabolism of several important drugs (phenytoin, tolbutamide, ibuprofen, and warfarin) (26) including anesthetic propofol.

### CYP2B6 Gene

The gene for CYP2B6, encodes a number of cytochrome P450 enzyme superfamilies (CYP2A, CYP2B, and CYP2F). These enzymes belong to monooxygenases, catalyze reactions in the synthesis of cholesterol, steroids, and other lipids, as well as biotransformation reactions of many drugs, including propofol. Enzymes are localized in the endoplasmic reticulum. Phenobarbital is known to strongly induce the synthesis of these enzymes, while clopidogrel inhibits it (27). Families of this enzyme participate in the metabolism of xenobiotics, such as chemotherapeutics (cyclophosphamide and ifosphamide), anti-inflammatory drugs, anesthetics and benzodiazepines. The gene for CYP2B6 is located on chromosome 19 (19q13.2), contains 9 exons, encodes a 48-kDa protein composed of 491 amino acids. The CYP2B6 gene contains over 28 alleles and over 100 SNPs and is considered a highly polymorphic P450 gene. A special variant of the CYP2B6*18 gene [I328T], predominantly present in Africans (4–12%) does not express proteins (enzymes). The CYP2B6 gene polymorphism is of particular importance in the treatment of HIV patients treated with a reverse transcriptase inhibitor (efavirenz), for whose metabolism this enzyme is responsible (28). The c.516G > T SNP allele variant in exon 4 (rs3745274) is responsible for reducing the amount of functional transcript for a given enzyme (29). Age, sex, nutritional status, disease state, drugs, and a patient’s heredity all influence the expression of CYP enzymes.

### UGT1A9 Gene

The UGT1A9 gene belongs to the family of genes responsible for the synthesis of the enzyme UDP-glucuronyl-sulfo-transferase. These enzymes participate in the glucuronidation reaction, in which glucuronic acid is conjugated to one of many different substances. In addition to the liver, enzymes are also present in the kidney, colon, ovary, testis and skin. The gene for UGT1A9 is located on chromosome 2 (q37.1). The gene locus comprises 13 unique alternative exons, of which the first 4 are considered pseudogenic, show significant variability, and encode a site in the substrate binding enzyme. UGT1A9 and UGT2B7 gene products participate in the second phase of biotransformation of endogenous and exogenous substrates. Variants of the UGT1A9 allele (c.98T > C as well as 766G > A) are the most common polymorphic forms that occur predominantly in the inhabitants of Asia and America. The gene for UGT1A9 is highly polymorphic (30), so carriers of some allelic variants can potentially face the described side effects, which leads to a significant reduction in the expression of the gene itself and, as a consequence, a decrease in glucuronidation of metabolites.

### Effects of CYP2C9, CYP2B6, and UGT1A9 Genotypes on Propofol Pharmacokinetics

Eugene was the first to propose genotype-based dose modifications for patients who were administered propofol (31). The final PK (parametric pharmacokinetics) analysis covered 51 participants in total. The propofol concentration-time data was characterized using a two-compartment gamma multiplicative error model. The UGT1A9 and CYP2B6 G516T gene variants did not result in statistically significant differences in PK parameters, while the CYP2B6 A785G gene variants did result in statistically significant differences in elimination rate, especially in older patients. If no dosage adjustment is done, the CYP2B6 AA and AG patients will be exposed to roughly 250 percent greater blood propofol levels in a brief 1-h infusion, according to modeling and simulation. Because the maintenance infusion dose is proportional to the clearance rate, precision guided dose adjustments for the CYP2B6 AA and AG genotypes necessitate a 50% reduction in infusion dose to 25mg/kg/min, as indicated and demonstrated in the results (31).

Loryan et al. (32) evaluated common CYP2B6 and UGT1A9 SNPs in propofol patients, but no significant genotype-based findings were discovered. Similarly, Choong et al. (33) observed that women metabolize propofol faster than males despite no significant differences in CYP2B6 or UGT1A9 SNPs on propofol metabolism. However, through a functional estrogen response element in the upstream regulatory CYP2B6 sequences, estrogen receptors have been demonstrated to boost CYP2B6 gene expression (34). As a result, it’s possible that sex hormone levels are a possible cause of the observed sexual dimorphism in propofol metabolite formation (33).

Several investigations have sought to see if the polymorphism CYP2B6 gene causes any substantial changes in propofol clearance and awareness after bolus doses and infusions, but none have resulted in gene-guided propofol dosage adjustments (35–38). Fujita et al. (39) investigated whether sex and cytochrome P450 (CYP) 2B6 and UDP glucuronosyltransferase (UGT) 1A9 polymorphisms influenced the discrepancy between predicted and measured plasma propofol levels during 4 h of target-controlled infusion (TCI). According to the authors, the propofol TCI system is more accurate in women than in men. Also, women’s plasma concentrations of propofol decline more quickly than men’s, and women recover from propofol anesthesia faster than males (39). This sex difference could be related to the fact that women’s livers have 1.9-fold higher CYP2B6 protein levels than men’s (32). On the other hand, Fujita et al. (39) concluded that the discrepancy between the predicted and actual plasma propofol concentrations in the perioperative period with continuous propofol infusion for 4 h was unaffected by CYP2B6 and UGT1A9 polymorphisms. Using stepwise multiple linear regression analysis to detect important factors of propofol pharmacokinetics in 94 patients (51 males, 43 females) who underwent lung surgery with total intravenous anesthesia (TIVA), Kobayashi et al. (37) concluded that sex differences, CYP2B6 polymorphisms, but not UGT1A, influenced propofol pharmacokinetics.

Kanaya et al. (40) found that body mass index (BMI) had an effect on propofol pharmacokinetics after a single intravenous dosage, whereas UGT1A9 and CYP2B6 SNPs, other clinical parameters, and hemodynamic variables had no effect. These findings suggested that BMI is a separate factor that influences propofol pharmacokinetics (40).

Mikstacki et al. (41) wanted to verify if the genetic mutations c.516G > T in the CYP2B6, c.98T > C in the UGT1A9, and c.1075A > C in the CYP2C9 genes had any effect on the individual propofol pharmacokinetic profile in Polish individuals having general anesthesia. A total of 85 patients were enrolled in the research. Rapid metabolizers were statistically more likely to be homozygotes c.516 T/T in the CYP2B6 gene. The pharmacokinetic profile of propofol was not affected by SNPs c.98T > C in the UGT1A9 and c.1075A > C in the CYP2C9 genes. The mean propofol retention time (MRT) was shown to be linked with the patient’s BMI. According to Mikstacki et al. (41) only the polymorphism c.516G > T in the CYP2B6 gene and BMI have an effect on propofol metabolism and may have a role in propofol anesthesia optimization.

A total of 138 propofol-treated patients were enrolled, and environmental, clinical, and surgical data were gathered by Mourão et al. (38). The length of the surgery and the weight of the patient raised the propofol dose, whereas age and the presence of the T allele reduced the total dose of the medicine required. The total propofol dosages were 151.5 64.2 mg and 129.3 44.6 mg, respectively, based on the GG or GT/TT genotypes (*p* = 0.043). According to their findings, these factors account for 34% of the variation in the needed propofol dose, and the CYP2B6 c.516G > T polymorphism, which slows drug metabolism, contributes for about 7% of the drug dosage. Mastrogianni et al. (36) showed a substantial correlation between the CYP2B6 G516T variant and high blood propofol concentrations after a single bolus dosage.

The goal of Pavlovic et al. (25) study was to determine how UGT1A9 98T > C, CYP2B6 516G > T, and CYP2C9 430C > T genetic polymorphisms affected propofol pharmacokinetics in children of different sexes and ages who underwent total intravenous anesthesia (TIVA) and deep sedation during diagnostic and therapeutic procedures. This prospective study included 94 children aged 1–17 years old with an ASA I-II status who underwent a conventional anesthetic regimen for TIVA, which included continuous propofol administration. The results indicated that UGT1A9 genotype is an independent predictor of propofol concentration in children 10 min after the end of the continuous infusion. The propofol distribution constant was greater in carriers of the polymorphic UGT1A9 C allele. The polymorphic CYP2B6 T allele carriers received a considerably lower overall and first propofol dose. Unlike the UGT1A9 gene polymorphism, the investigated CYP2C9 and CYP2B6 gene polymorphisms are not independent predictors of propofol pharmacokinetics.

Khan et al. (42) concluded that patients with UGT1A9–331C/T had a greater propofol clearance and required a higher propofol induction dose. Patients with UGT1A9–1818T/C took longer to lose consciousness, while those with CYP2C9*2/*2 had higher propofol plasma concentrations than the others (42). Takahashi et al. (43) reported that the D256N polymorphism in UGT1A9 lowers enzyme activity in an *in vitro* study, suggesting that carriers of D256N may be at risk of propofol side effects.

### ABCB1 Gene

The ABCB1 (MDR1, P-gp) gene was the first ABC transporter to be discovered and studied. This gene produces a transmembrane protein that facilitates ATP-dependent molecular transport (44). The P-gp protein is expressed on the luminal surface of blood-brain barrier (BBB) capillary endothelial cells and is known as the “guardian” of the brain. The P-gp transporter at the blood–brain barrier prevents active efflux of drugs into the CNS. It also allows harmful substances to be transported out of the brain (45). The absorption, distribution, and bioavailability of anesthetic drugs might be affected by variations in genes encoding P-gp protein. The MDR1 or ABCB1 gene, which encodes this transporter protein, has multiple functional polymorphisms, including 1236C > T, 2677G > T/A, and 3435C > T, which have been linked to anesthetic drug response variability (46). The c.3435C > T variant in exon 26 is one of over 100 polymorphic variants of this gene that have been found so far. This polymorphism has been linked to changes in P-gp expression and medication response in a variety of clinical settings (47).

According to the findings of Ivanov et al. (48) the ABCB1 (c.3435C > T) variation has no effect on clinical parameters in propofol patients. Although there is limited information on the impact of this variant on propofol therapy, their findings are consistent with those of Zakerska-Banaszak et al. (49), who found no statistically significant differences between propofol therapeutic effects and ABCB1 gene variants. Wolking et al. (50) pointed out that the genetic impact of ABCB1 polymorphisms on P-gp transporter expression and/or function is unclear and for that reason. for patients with the ABCB1 gene variation, no changes in drug dose or therapeutic substitution have been indicated (50). On the other hand, the mutation in ABCB1, SNP c.1236 C > T (rs1045642), was partly the reason for differences in the anesthetic effects when propofol was combined with remifentanil for pediatric tonsillectomy as reported by Zhang et al. (51). Liew et al. (52) used the databases PubMed, Medline, and Ovid to conduct a systematic search of the literature. The search was restricted to publications published between 2006 and 2020. In order to extract relevant papers from the databases, search phrases such as gene polymorphism, MDR1, ABCB1, opioid, propofol, children, pain, anesthetic, anesthesia, analgesic, analgesia, odds ratio, and surgery were used. For the analysis and summaries, a total of 2,554 patients from 17 papers were considered. The papers selected focused on the impact of SNPs in the ABCB1 gene (1236C > T, 2677G > T/A, and 3435C > T) on anesthetic and analgesic effects. Based on the evidence, genetic polymorphism in the ABCB1 gene had a substantial impact on anesthetic effects (mutational homozygous TT genotype in both ABCB1 1236C > T and 3435C > T was linked with a reduced anesthetic effect) but no apparent impacts on analgesia (51, 53).

## Pharmacodynamics of Propofol – Mechanism of Action

Propofol, like other intravenous anesthetics like benzodiazepines and barbiturates, acts by activating the central inhibitory neurotransmitter gamma-aminobutyric acid (GABA) to produce hypnosis (54). Gamma-aminobutyric acid type A (GABAA) receptors are ligand ion channels composed of different subunits (α, β, γ, δ, ε, θ, ρ, π) that form a pentameric structure containing a central chloride channel. Binding of propofol molecules to the receptor leads to increased influx of chloride ions and hyperpolarization of neurons, which leads to non-response to external stimuli. Propofol appears to be less effective at receptors containing β 1 than at those containing β 2 or β 3 subunits (55). Propofol also affects the presynaptic mechanisms of GABA transmission, such as GABA uptake and release (56). Its site of action appears to be different from that of barbiturates and benzodiazepines. The effect of propofol on other receptors has not been established with certainty. It has a very solid antiemetic effect (possible anti-serotonergic effect). It probably activates inhibitory glycine receptors at the spinal cord level, and inhibits nicotinic acetylcholine receptors, as well as excitatory glutamate NMDA and AMPA receptors.

The inter-patient variability in the propofol dose necessary to achieve BIS < 70, as well as the estimated apparent systemic clearance and “time to eye opening” following TIVA, was studied by Iohom et al. (57). Although it appeared that genetic variants in the CYP2B6 and GABAA(ε) genes might explain for some of this variation *in vivo*, the genetic variants studied did not account for the majority of it. Ivanov et al. (48) found differences between the given propofol doses in patients with different genotype for the polymorphism studied i.e., in *GABRA1* (c.1059 + 15G > A) carriers the initial, additional, and total dose of propofol decreased with age (*p* > 0.05). GABRA2 rs35496835, GABRB1 rs1372496, GABRG2 rs11135176, GABRG2 rs209358, GAD1 rs3791878, SLC1A3 rs1049522, and gender were all revealed to be significant predictors of loss of consciousness latency following propofol administration (58). Blood pressure decrease during anesthesia induction was highly linked with GABRA2 rs11503014. Because there was no direct evidence of a link between hypotension and GABA, Zhang et al. (58) pointed out that future research should focus on the mechanisms underlying the effects of GABAAR gene polymorphisms on blood pressure during TIVA with propofol.

## Clinical Utility of Pharmacogenomic Testing in Anesthesiology

The patient’s most vulnerable period is during the perioperative period, and personalized anesthetic approach will be the future standard. However, there is currently no clear clinical data addressing the efficacy and cost–effectiveness of pharmacogenomic testing for the majority of drugs (59, 60).

Over the past few years, the link between pharmacogenetics and anesthesiology has become even stronger due to the emergence of new data on the effects of genetic variations on the pharmacokinetics and pharmacodynamics of used drugs. Frequent conflicting results between studies make it difficult to include pharmacogenetics in anesthesiology (61). There are currently no clinically relevant guidelines in the field of anesthesiology for the individualization of the use of general anesthetics based on conducted pharmacogenetic analyzes of biological material in patients. From a scientific and professional point of view, this is especially important in anesthesiology because anesthesiologists could be considered as practicing pharmacologists and the use of total intravenous anesthesia (TIVA) using propofol is just a good example of “pharmacology in action” (62).

Since the Enhanced Recovery After Surgery (ERAS) practices emphasize a multidisciplinary comprehensive approach to the care of surgical patient (63) the most recent elements of ERAS involve drug administration and the feasibility of introducing pharmacogenomics testing in ERAS to guide drug administration (64).

Overall, there are two types of obstacles to clinical pharmacogenomic testing implementation: first, determining whether the testing should be done at all, based on the availability of evidence and cost-effectiveness, and second, overcoming challenges associated with integration into the clinical system and work flow (such as clinical labs’ struggle to comply with regulatory frameworks designed for non-genetic or single-gene tests). Additionally, initial pharmacogenetic studies have concentrated on one gene and one drug, as is usual in genetic research. However, extrapolating data from one gene to a single drug is difficult due to the fact that drugs are rarely used alone and that the drug response pathway normally involves more than one gene (61). Furthermore, due to statistical power, tests are frequently repeated, and patients with lower frequency allele subgroups are constantly under-represented, resulting in potentially incomplete results. Besides all that, despite historically being the physician champions for pharmacogenetic testing to guide perioperative care, anesthesiologists sometimes had to rely on the other specialties to order the test (65). Nevertheless, further research is needed to define and describe polymorphic enzymes in order to better understand interindividual variations in the glucuronidation metabolic pathway, as well as their pharmacological and toxicological side effects.

Although positive pharmacogenetic polymorphic associations with clinical significance have been discovered, there is a lack of reproducibility because most studies focus on single variant associations, whereas interindividual differences in propofol metabolism may be best explained by the contribution of multiple pathways. Indeed, the perioperative phase has a high risk of serious adverse reactions due to the narrow therapeutic index and great variability in patient responses to anesthesia and surgery. Additional metabolites must be identified in order to validate xenobiotic exposure in a larger detection window, particularly in different samples. Besides, regardless of the fact that there are sex and racial/ethnic differences in propofol response, there is no strong evidence linking genetic variation to such findings, possibly due to the additional influence of weight, height, and lean body mass, environmental factors, and severe hepatic or renal impairment on propofol pharmacokinetics (17, 66).

Finally, propofol metabolomics has not yet been thoroughly investigated (8), and more research is needed to determine whether the various metabolomic patterns are clinically significant, taking into account sex, age, genetic polymorphisms, and other factors such as comorbidities.

## Conclusion

There are many unresolved questions regarding the importance of pharmacogenetic studies in anesthesiology. In recent years, there has been a significant breakthrough in this type of research, which has been largely limited to one or a group of genes. In addition, the role of a number of well-known factors such as age, gender, associated diseases, BMI, type of surgery is unambiguous, so that our obtained results of pharmacogenomic studies can often confuse or lead to wrong conclusions. The value of pharmacogenomics in anesthesia has been firmly demonstrated throughout history, but whether it has a place in everyday clinical practice at this moment needs to be determined. What is certain is that we will have to wait for more solid evidences from future studies and projects.

## Author Contributions

IB wrote and revised the manuscript. TJ contributed to the idea generation and edited the final manuscript. DP, VM, and ID searched the literature and revised final version. MS and DS reviewed and revised final version of the manuscript. All authors have read and approved the manuscript.

## Conflict of Interest

The authors declare that the research was conducted in the absence of any commercial or financial relationships that could be construed as a potential conflict of interest.

## Publisher’s Note

All claims expressed in this article are solely those of the authors and do not necessarily represent those of their affiliated organizations, or those of the publisher, the editors and the reviewers. Any product that may be evaluated in this article, or claim that may be made by its manufacturer, is not guaranteed or endorsed by the publisher.
